# Protocol for a cluster randomised controlled trial of an intervention to improve the mental health support and training available to secondary school teachers – the WISE (Wellbeing in Secondary Education) study

**DOI:** 10.1186/s12889-016-3756-8

**Published:** 2016-10-18

**Authors:** Judi Kidger, Rhiannon Evans, Kate Tilling, William Hollingworth, Rona Campbell, Tamsin Ford, Simon Murphy, Ricardo Araya, Richard Morris, Bryar Kadir, Aida Moure Fernandez, Sarah Bell, Sarah Harding, Rowan Brockman, Jill Grey, David Gunnell

**Affiliations:** 1 School of Social and Community Medicine, University of Bristol, Canynge Hall, 39, Whatley Road, Bristol, BS8 2PS UK; 2 DECIPHer, School of Social Sciences, Cardiff University, 1-3 Museum Place, Cardiff, CF10 3BD UK; 3 Bristol Randomised Trials Collaboration, School of Social and Community Medicine, University of Bristol, Canynge Hall, 39, Whatley Road, Bristol, BS8 2PS UK; 4 University of Exeter Medical School, South Cloisters, St Luke’s Campus, Exeter, EX1 2LU UK; 5 London School of Hygiene and Tropical Medicine, Keppel Street, London, WC1E 7HT UK

**Keywords:** Cluster randomised controlled trial, Secondary school, Mental health, Wellbeing, Teacher, Adolescence

## Abstract

**Background:**

Teachers are reported to be at increased risk of common mental health disorders compared to other occupations. Failure to support teachers adequately may lead to serious long-term mental disorders, poor performance at work (presenteeism), sickness absence and health-related exit from the profession. It also jeopardises student mental health, as distressed staff struggle to develop supportive relationships with students, and such relationships are protective against student depression. A number of school-based trials have attempted to improve student mental health, but these have mostly focused on classroom based approaches and have failed to establish effectiveness. Only a few studies have introduced training for teachers in supporting students, and none to date have included a focus on improving teacher mental health. This paper sets out the protocol (version 4.4 20/07/16) for a study aiming to address this gap.

**Methods:**

Cluster randomised controlled trial with secondary schools as the unit of randomisation. Intervention schools will receive: i) Mental Health First Aid (MHFA) training for a group of staff nominated by their colleagues, after which they will set up a confidential peer support service for colleagues ii) training in MHFA for schools and colleges for a further group of teachers, which will equip them to more effectively support student mental health iii) a short mental health awareness raising session and promotion of the peer support service for all teachers. Comparison schools will continue with usual practice. The primary outcome is teacher wellbeing measured using the Warwick Edinburgh Mental Wellbeing Scale (WEMWBS). Secondary outcomes are teacher depression, absence and presenteeism, and student wellbeing, mental health difficulties, attendance and attainment. Measures will be taken at baseline, one year follow up (teachers only) and two year follow up. Economic and process evaluations will be embedded within the study.

**Discussion:**

This study will establish the effectiveness and cost-effectiveness of an intervention that supports secondary school teachers’ wellbeing and mental health, and improves their skills in supporting students. It will also provide information regarding intervention implementation and sustainability.

**Trial registration:**

International Standard Randomised Controlled Trial Number: ISRCTN95909211 registered 24/03/16

**Electronic supplementary material:**

The online version of this article (doi:10.1186/s12889-016-3756-8) contains supplementary material, which is available to authorized users.

## Background

### Teacher mental health and wellbeing

Teachers are consistently reported to be at increased risk of common mental health disorders compared to other occupations [1, 2]. Findings from the WISE pilot study [3] showed secondary school teachers scored approximately 0.5 standard deviations lower than the general working population on the Warwick Edinburgh Mental Wellbeing Scale (WEMWBS) [4]. Further, 19.4 % of the sample scored as moderately to severely depressed on the 9 item Patient Health Questionnaire (PHQ-9) compared to a general population prevalence of 8–10 % [5, 6].

Failure to attend to heightened levels of stress and distress may lead to longer term mental health problems, poor performance at work (presenteeism), sickness absence, and health-related retirement in teachers [7–9]. This has implications not only for teachers’ own health, but for the quality of staff-student relationships and for student health. Teachers with poor mental health have been reported to find it difficult to manage classes effectively, and to develop supportive relationships with students [10]. Difficult teacher-student relationships in secondary school have been found to predict psychiatric disorder and exclusion from school three years later [11]. Conversely, supportive teacher-student relationships predict lower student depression, and higher student classroom engagement, leading to higher achievement [12, 13]. Young people in the UK have amongst the worst wellbeing in Europe [14] with almost 10 % having a clinically diagnosed mental health condition [15], and as many as 18 % of 16–17 year olds engaging in self-harm behaviour [16]. Teachers are the professionals who have the most contact with children and young people regarding mental health issues [17], and therefore are in a prime position to provide support. However, where teachers experience poor wellbeing this reduces their belief that they can help students with emotional or behavioural problems [18]. Further, teachers report a lack of training in how to support such students effectively [19, 20], which is a source of stress in itself.

### School-based mental health interventions

A number of school interventions have focused on improving student mental health in recent years, but these have primarily used classroom based psychological or educational programmes. Two systematic reviews found that such interventions had modest effects on reducing student depression and anxiety, but that more evidence is needed beyond small efficacy studies [21, 22]. One large scale implementation study recently conducted in the UK, aiming to reduce depression among secondary school students using cognitive behavioural therapy, found no effects, with the authors concluding that there is insufficiently strong evidence to support such universal, classroom-based approaches [23]. Fewer studies have focused on intervening in aspects of school life beyond curriculum content. Two high quality randomised controlled trials evaluated the impact of a ‘whole school approach’ on student mental health - in which attempts were made to change the school climate alongside curriculum content - neither of which found positive results [24, 25]. It has been suggested that designing a very broad and flexible approach, such as that taken in these studies, creates too much variability in intervention implementation across schools, which may limit effectiveness [26]. Interventions may be more effective if they focus on changing a smaller number of more specific components of school life that are known to be important to mental health.

As noted above, two potential areas for intervention which may improve both teacher and student mental health are increasing support for teachers themselves and developing teachers’ skills in supporting students. The Incredible Years Teacher Classroom Management programme focuses on improving teacher support for positive student behaviour through increasing their skills in supportive classroom techniques within primary schools [27]. Pilot trials in the UK have reported promising results in terms of reduced negative behaviours among children with poorer mental health, a reduction in teachers’ negative behaviours towards such children, improved teacher self-efficacy and improved teacher emotional health [28, 29]. No randomised controlled trials (RCTs) have examined such an intervention at secondary school level. However, two studies have evaluated the effect of delivering mental health training to secondary school teachers. The SEYLE study, a three arm cluster RCT across ten European countries, compared the effectiveness of training teachers to recognise and support students at risk of suicide, with i) raising student awareness about mental health and suicide and ii) screening by professionals [30]. Only the student training intervention showed statistical evidence of an impact on suicide ideation and attempts, and the authors suggest that the ineffectiveness of the teacher training element may have been due to poor teacher wellbeing reducing their ability to support students. An earlier RCT evaluated the impact of delivering youth Mental Health First Aid (youth MHFA) training to school staff [31]. MHFA was developed in Australia with the aim of equipping lay people to recognise the signs and symptoms of common mental health problems [32]. Youth MHFA has the same aims but is targeted at individuals who work with teenagers. This study found positive changes in staff mental health knowledge, attitudes and confidence in helping, but no change in reported helping behaviours or student mental health [31]. However, as with the SEYLE study, there was no intervention to support the mental health of the teachers themselves.

In summary, there is a need to develop and evaluate secondary school mental health interventions that focus on both teacher mental health and wellbeing, and the support that teachers are equipped to provide to students. We piloted such an intervention in a small cluster randomised trial (*n* = six schools) where: i) a group of secondary school staff were trained in standard Mental Health First Aid (MHFA), and set up a confidential peer support service for their colleagues ii) a further group of staff within the same school received youth MHFA training [33]. The intervention was found to be feasible and acceptable to schools, although suggestions for improvement were made: reducing the length of the youth MHFA training and targeting tutors and classroom teachers to attend, ensuring the peer support service was promoted sufficiently and supported by senior leaders, and making certain that peer supporters were not overloaded with the extra responsibility. The pilot study also enabled us to test the feasibility of key aspects of the trial design. We found that schools from a range of socioeconomic catchment areas were able to be recruited and retained, it was possible to collect self-report measures of staff and student wellbeing and mental health, and staff presenteeism and absence, and high response rates were achieved if data collections were organised during staff meetings/class time. Following the Medical Research Council’s (MRC) framework for the evaluation of complex interventions [34], we concluded that it would be feasible and justifiable to conduct a full RCT of the intervention, to evaluate its impact on teacher and student outcomes.

## Methods/Design

### Study aim

To evaluate the effectiveness and cost effectiveness of an intervention that provides peer support for secondary school teachers, and teacher training in Mental Health First Aid (MHFA) for Schools and Colleges, using an RCT design with embedded process and economic evaluations.

### Primary objective

To establish if the WISE intervention leads to improved teacher emotional wellbeing compared to usual practice.

### Secondary objectives

To address the following research questions:

1. Does the WISE intervention lead to lower levels of teacher depression, absence and presenteeism, improved student wellbeing, attendance and attainment, and reduced student psychological difficulties compared to usual practice?
2. Do any effects of the intervention differ according to geographical area and the proportion of children receiving free school meals (FSM – an indicator of the deprivation of the catchment area) in a school, or according to individual level baseline mental health, gender, ethnicity and FSM (for teacher and student outcomes)?
3. What is the cost of the WISE intervention, and is it justified by reduced teacher absenteeism/presenteeism or improvements to teacher wellbeing and other teacher and student outcomes?
4. Does the WISE intervention work according to the mechanisms of change hypothesised in the logic model?
5. Is the WISE intervention sustainable?

### Study design

This study is a cluster RCT, with embedded economic and process evaluations. Schools are the unit of allocation because the intervention is school-wide. There will be two study sites, one in South West England and one in Wales, which will enable consideration of the generalizability of the findings.

In Wales, eligible schools will be drawn from two administrative areas (consortia) (*n* = 88), and within each area will be stratified into three levels according to FSM (high, medium and low). Two schools will be randomly selected from each stratum in each area, giving a total of 12 schools. A relevant senior manager in each school, e.g., a deputy head in charge of pastoral care, will be approached by the study team and their school invited to participate in the study. Any schools that decline will be replaced by a randomly selected school from the same stratum and geographical area.

In England, the study will be advertised by local public health teams to senior leaders and healthy schools contacts at all eligible schools within a 30 mile radius of one city (*n* = 64), and schools will then be followed up by the study team. Those who express interest in participating will be grouped by administrative area (city local authority [LA]/LA outside the city) and stratified into three levels according to FSM (high, medium and low). The aim will be to recruit two schools per stratum in each area, so if a stratum has more than two interested and eligible schools within the time frame, two will be randomly selected to give a total of 12 schools.

The sampling frame includes two administrative areas in each site to ensure a large enough sample is obtained. Schools will be stratified by area and FSM to ensure balance across study arms, in case administrative area or FSM status is associated with mental health outcomes. Including schools from different areas and with different levels of FSM eligibility will also help ensure the generalisability of the findings. If difficulties are encountered due to lack of interested or eligible schools in a particular stratum, two strata will be merged for that area. Table 1 shows the planned distribution of schools by strata, area and site.

**Table 1:** Stratification of participating schools

		England		Wales	
		Area 1	Area 2	Area 3	Area 4
FSM eligibility (%)^a,b^	Low	2 schools	2 schools	2 schools	2 schools
		(range: 8.4–17.2)		(range: 3.8–15.3)	
	Medium	2 schools	2 schools	2 schools	2 schools
		(range: 18.5–34.9)		(range: 16.1–25.1)	
	High	2 schools	2 schools	2 schools	2 schools
		(range: 39.2–77.0)		(range: 25.8–44.8)	

Notes

^a^Cut-offs for each FSM stratum were based on national mean and distribution, and therefore were slightly different for England and Wales (mean = 29.4 % over the past 6 years for England and 17.0 % over the past 3 years for Wales)

^b^Range given is the range of FSM among eligible schools in each strata

Many schools in England are Academies (state-funded but independent of local authority control), some of which are linked administratively in so-called academy chains. In order to avoid potential contamination at this administrative level, once one school from an academy chain has been selected, any others from the same chain and administrative level, will be excluded from the remaining selection process.

Selected schools will be screened to ensure the training package being used in the intervention (MHFA) has not previously been delivered within the school. Schools that are receiving or have received MHFA training will be excluded and replaced by another school randomly selected from the same stratum and geographical area.

### Study population

The population from which the 24 schools in our sample will be recruited is secondary schools in the two geographical areas. Our study population is all teaching staff in those schools, and students in year 8 at baseline (12–13 year olds) and year 10 (14–15 year olds) at the second follow up. This year group was selected as they will be in year 10 at the two year follow up, and therefore still in school, whereas those who are in years 9–11 at baseline may have finished school by the two year follow up. The wellbeing measure used (see below) has not been validated for students younger than year 8. We assume that any effect on this year group will be indicative of all students as this is a school-wide intervention. Each school will have approximately 60 teachers and 150 year 8 students, depending on size.

### Inclusion criteria

All state mainstream non-fee paying secondary schools within the relevant geographical areas.

### Exclusion criteria

The following schools will be excluded from the sampling frame:

- Fee paying schools
- Special schools (e.g., for those with learning disabilities)
- Pupil referral units
- Schools that took part in the pilot study and feasibility work
- Schools already participating in other, similarly intensive, research studies
- Schools already receiving MHFA training or other similar interventions such as mindfulness training
- Schools without available free school meal eligibility data (e.g., because they have recently merged)

### Randomisation

Allocation will take place after baseline measures have been collected, and will be conducted by the study statistician, blinded to any identifying information. The statistician will randomly allocate schools stratified by geographical area and FSM scores (see Table 1) to intervention or control arm, using computer-generated random numbers.

### Blinding

It is not possible for study participants to be blind to intervention status, or for the research team leading the outcome data collections as they will also have organised intervention delivery with the schools and conducted the process evaluation. However casual staff assisting with data collection and inputting the questionnaire data, the statisticians analysing the primary and secondary outcome data and the health economists undertaking the economic analysis will be blind to intervention status.

### Consents

Consent for the school to participate will be gained from the head teacher by the study team, and a written agreement signed by them or someone they have appointed (Additional file 1). All teacher and student participants will be given information sheets at least 2 weeks before each outcome data collection session, outlining the right to opt out of participation, the purpose of the study, potential benefits or harms in taking part, where the data collected will be kept, and what it will be used for. Those not wishing to take part in the data collection will be advised to return the blank questionnaire. Letters containing the same information will be posted or emailed by schools to all parents at least 1 week before student data collection, accompanied by opt out forms to be returned to the study team (Additional file 2). Any student whose parent returns the opt out form will not take part in the questionnaire data collection.

For the process evaluation, information will be provided to all staff and students invited to take part in an interview or focus group at least 2 weeks before, and written consent to participate will be obtained by the study team (Additional file 3). Parental written consent will also be obtained for students invited to take part in a focus group.

### Intervention

The intervention has three elements:

1. **Staff peer support service:** all staff in the intervention schools will be invited to nominate colleagues whom they consider would make good peer supporters, via a confidential, anonymous written questionnaire. The one exclusion criterion for nomination will be membership of senior management, as pilot findings indicated that staff might feel uncomfortable using a support service that included senior leaders. The 8 % of staff with the most nominations - ensuring a mix of gender and teaching/non-teaching role by moving down the list until this mix has been met – will be invited to attend the 2 day standard MHFA training and to then set up a confidential peer support service for colleagues to access as and when required, with the aim of providing a listening ear and signposting to other services as appropriate. Anyone invited who does not want to do this will be replaced by the person matching their gender and role with the next highest number of nominations, and numbers of individuals not consenting to take part will be noted. The peer support service will be advertised through an initial awareness raising event and refresher one year later (see point 3), and also through posters, staff emails, and at staff meetings.
2. **MHFA for Schools and Colleges training for teachers:** A minimum of 8 % of all teaching staff (up to a maximum of 16) who have pastoral duties such as tutors or heads of year will receive this 1 day training, which is based on the youth MHFA course, but targeted to meet the needs of educational environments. Trained teachers will then continue with their usual teaching and pastoral roles within school, but will apply the MHFA for Schools learning in their day to day interactions with students; responding to signs and symptoms of distress and providing initial help and support to individuals they identify as at risk of mental health difficulties.
3. **Mental health awareness raising session for all teachers:** All teaching staff will receive a one hour awareness raising session, which will introduce the peer support service and focus on the importance of mental health issues in schools. A refresher session will be delivered at the start of the next academic year by the peer supporters themselves, to ensure that the profile of the peer support service is maintained.

The MHFA training will be delivered by Healthy Schools Coordinators in Wales who have completed an MHFA instructor course, and by external MHFA trainers in England. All trainers will have been quality assessed by MHFA England and will be accredited trainers.

The comparison schools will continue with usual practice in terms of teacher support and training, the details of which will be examined as part of the process evaluation.

### Outcome measures

The outcome measures will be collected at baseline and at the one year follow up (teacher outcomes only) and two year follow up (teacher and student outcomes). Baseline and outcome data will be collected during the final term of the academic year in each case. Table 2 shows the outcomes to be collected at each time point (primary outcome is in bold).

**Table 2:** Outcomes to be collected by time point

	Baseline	Year 1	Year 2
Teacher	WEMWBS PHQ-8 Presenteeism Teacher absence (past 28 days) Teacher absence (%) [school level] Teacher retirements and resignations [school level]	WEMWBS PHQ-8 Presenteeism Teacher absence (past 28 days) Teacher absence (%) [school level] Teacher retirements and resignations [school level]	WEMWBS PHQ-8 Presenteeism Teacher absence (past 28 days) Teacher absence (%) [school level] Teacher retirements and resignations [school level]
Student	WEMWBS SDQ Attendance (%) [school level – all year groups] Attainment (%) [school level - year 11]		WEMWBS SDQ Attendance (%) [school level - all year groups] Attainment (%) [school level - year 11]

#### Primary outcome

The primary outcome is teacher wellbeing. This will be measured via self-report questionnaires during staff meetings, using the WEMWBS [4]. The WEMWBS is a comprehensive (incorporating elements of both subjective and psychological wellbeing) 14-item survey, responsive to change and validated among community samples of adults in the UK. It will be measured as a continuous variable. Scores range from 14 to 70.

#### Secondary outcomes – teachers

Teacher depression, absence and presenteeism will be measured within the same self-report questionnaire. Depression will be measured using the 8 item Patient Health Questionnaire (PHQ-8) [5]. The PHQ-8 is suitable for measuring levels of depressive symptoms in population-based studies and is short enough to be used in self-report surveys. Unlike the 9-item version it does not contain a question about thoughts of self-harm – due to such a low number of participants in the pilot reporting suicidal thoughts, it was decided that measuring suicidality as an outcome was not necessary. Sickness absence and presenteeism during the last 4 weeks will be measured using questions adapted from the Work Productivity and Activity Impairment questionnaire (WPAI) [35]. Teacher absence days, retirement and resignations will also be measured at one and two years using routine data collected by schools.

Teacher PHQ-8 will be measured as continuous variable, and as an ordinal variable (a score of 0–4 indicating no depressive symptoms/5–10 indicating mild symptoms/10–14 indicating moderate symptoms/15–19 indicating moderately severe symptoms 20–24 indicating severe symptoms) and a binary variable, with a cut-point of 10 or more indicating depression [5]. Teacher absence and presenteeism will be treated as binary (any vs no absence in the previous four working weeks and health problems having 0 effect vs 1–10 effect on work over the previous four working weeks), and each variable will also be categorised and treated as ordinal, to examine whether the intervention has a differential effect on increasing levels of absence and presenteeism. Teacher absence, retirement/resignation and student attendance and attainment as reported by schools, will all be reported as a school level %.

#### Secondary outcomes – students

Student wellbeing will be measured using the WEMWBS, and psychological distress using the Strengths and Difficulties Questionnaire (SDQ), using a self-report questionnaire administered during class time. The WEMWBS has been validated among teenagers from thirteen years [36], and the SDQ is a widely used measure among this age group, with well-established norms for a UK population [37]. It is a 25-item scale measuring emotional and behavioural difficulties, with a self-report version for 11–16 year olds. Student attendance for all year groups (total %) and attainment for year 11 students (% achieving five or more GCSEs grades 5–9 including English and Maths) will be measured using routine school census data published online.

WEMWBS, SDQ, attendance and attainment will all be measured as continuous variables.

**Fig. 1:**
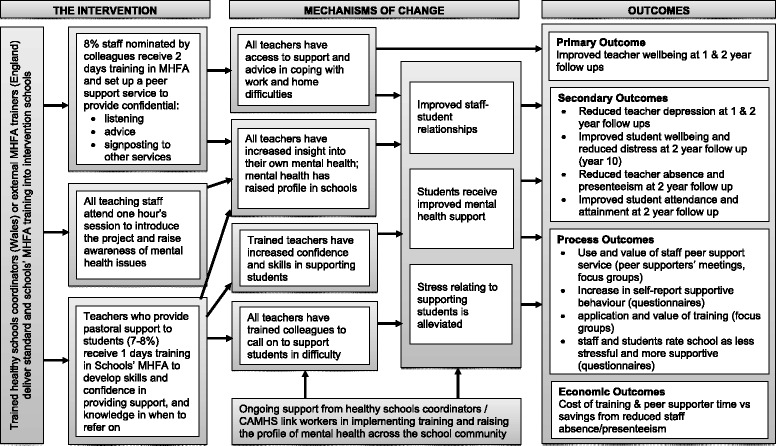
Logic Model of the WISE intervention

A logic model hypothesising the mechanisms by which the intervention will have an impact on the primary and secondary outcomes is shown in Fig. 1.

### Participant retention and loss to follow up

In the pilot study missing outcome data was largely due to participants not being present during data collection sessions. In this study we have built in time after the initial data collection sessions to follow up missing participants. Where possible second data collection sessions will be planned with students. It will be harder to ask schools to set aside more than one staff meeting for data collection, so teachers absent from the data collection session will be followed up by email and given the option of completing either an online survey or posting a paper one back to the study team. A certain number of participants will be lost to follow up due to leaving the school between baseline and follow up. For this reason our primary analysis will use a repeated measures design that includes all participants with any data (see below).

### Data management

Outcome data will be entered onto REDCap (Research Electronic Data Capture). Checks will be built into the database to disallow entry of impossible values. A randomly selected 10 % of the questionnaires entered will be checked by a second person to ensure data quality. Only the study team will have access to the final trial dataset. Information about each school and personal data from participants (names and teacher emails) will be stored in a database on a networked drive that is password protected and only accessible by the study team.

### Sample size calculation

The study is powered to detect a mean change of 3 points on the primary outcome - the WEMWBS score among teachers. This difference has been chosen as the minimum meaningful change discussed in a WEMWBS user guide [38]. A change of 3 points is also close to the difference in mean baseline scores between the highest and lowest ranked schools in the pilot study. Sample sizes were calculated using the clustersampsi command within Stata, which accounts for the clustered nature of the data by including intracluster correlation coefficients (ICCs) in the calculation. Based on the pilot data [34] we assumed a WEMWBS ICC of 0.01 (0.00, 0.05), a mean of 50 teachers followed up per school (with a coefficient of variation of sample size of 0.5), and a standard deviation for WEMWBS of 8.4. A sample size of 24 schools (12 intervention and 12 control) will achieve 83 % power at the 5 % significance level for an ICC of 0.05 (the highest of the confidence intervals in the pilot ICC); this would rise to 98 % for an ICC of 0.02.

### Statistical analysis

All statistical analyses will take account of clustering by school and repeated measures using random effect or robust standard errors for clustering and random effects for repeated measures. Analyses will be conducted using Stata (version 13 or higher). An intention to treat analysis will be applied.

#### Primary analysis

Repeated measures (random effects) models will be used to examine pattern of change in the primary outcome (teacher WEMWBS as a continuous measure) over baseline, one year and two year follow ups adjusted for stratification variables and additional appropriate variables. The models will include every teacher who has at least one measure (at baseline or either of the two follow-up periods), and are robust to data which are missing at random (MAR). Thus no imputation is needed for this analysis [39, 40]. Results will be presented as mean differences in the primary outcome between the trial arms, with 95 % confidence intervals and *p*-value.

#### Secondary analyses

Analysis will include, linear (continuous outcomes), multinomial (ordinal outcomes) and logistic (binary outcomes) regression models depending on the outcome measure. They will be used to compare the outcome at follow up by arm, initially unadjusted for variables (accounting for clustering), then adjusted for baseline outcome and for school-level FSM and geographical area, as stratifying variables [41]. Then finally adjusted for important covariates such as age, gender and any other covariates that are not balanced at baseline.

For individual level outcomes that are measured at 12 and 24 months (e.g., PHQ-8), repeated measures models will be used, including a random effect for each individual, and random effects to allow for clustering by school. All individuals with at least one observation of the outcome measure will be included in the model for that outcome measure, under a Missing at Random (MAR) assumption.

For individual level outcomes that are only measured at 24 months (e.g., student WEMWBS), a regression will be used, with robust errors to account for clustering.

For school level outcomes that are measured at both 12 and 24 months (e.g., teacher absence), repeated measures models will be used. For outcomes at school level which are only measured at 24 months (e.g., student attainment), a simple regression will be used.

#### Missing data

In the pilot study, missing data for teacher WEMWBS score was 1.6 % at baseline and 3.5 % at follow up, and for teacher PHQ-9 score was 7.4 % at baseline and 5.4 % at follow up. Missing data for student WEMWBS was 14.8 % at baseline and 10.9 % at follow up, and for student SDQ was 16.2 % at baseline and 12.7 % at follow up.

We will assess the impact of missing data and non-response on teacher WEMWBS and PHQ-8 outcomes and student WEMWBS and SDQ outcomes using an appropriate imputation model. If multiple imputation is used [42], imputation models will include all outcomes (baseline and follow-up), intervention arm, stratifying variables and a random effect for schools to allow for clustering, as well as any appropriate baseline covariates and interactions (see below). All secondary analyses will be repeated on the imputed datasets, and results combined using Rubin’s rules. We will also use sensitivity analyses to examine the impact of potential missing not at random (MNAR), by assuming that those with missing outcome scores had better/worse outcomes than equivalent individuals with complete data [43]. We will examine the sensitivity of our conclusions to missing outcome scores that are 20, 50 and 100 % SD worse than expected had they not been followed up, and similarly 20, 50 and 100 % SD better. We will also estimate the largest amount that would need to be added to/subtracted from imputed outcomes without changing the clinical interpretation of the trial.

#### Tests for interactions

Using appropriate interaction terms, we will test whether any effects of the intervention on teacher WEMWBS and PHQ-8 score differ according to: baseline teacher wellbeing/depression score (grouped as above or below the bottom quartile of the WEMWBS, or with a score of 10 or more on the PHQ-8), gender, geographical area (Wales/England) and school-level FSM. In the pilot less than 6 % of teachers selected an ethnicity other than white, and so ethnicity will not be considered in these analyses. We will look at the interaction between time and intervention, to test if the intervention had different effects at different time points. The effect of the intervention on student WEMWBS and SDQ score will be tested for interaction with baseline student wellbeing/SDQ score (grouped as above or below the bottom quartile of the WEMWBS and a score of 16 or more on the SDQ), gender, ethnicity, FSM eligibility, geographical area, and school level FSM. P-values will be interpreted with caution due to the low power and number of interactions being tested (e.g., we will use Bonferroni corrected or permutation *p*-values).

### Process evaluation

Informed by the Medical Research Council’s guidance [44], an embedded process evaluation will be conducted, using a combination of quantitative and qualitative methods. This will consider: mechanisms of change (see Fig. 1) and relevant contextual influences [45]; reach; contamination; intervention fidelity [46]; unintended harms [47]; acceptability; and sustainability.

Table 3 provides a summary of the ways in which the different process evaluation components will be assessed.

**Table 3:** Process evaluation components and relevant data

Evaluation component	Relevant data
Mechanisms of change	*Teacher questionnaires* Stress at school Satisfaction at school Help provided to colleagues and students Help received from colleagues School’s attitude to staff and student wellbeing Quality of relationships in school *Student questionnaires* Help received from teachers School’s attitude to student wellbeing Quality of relationships with teachers *Interview with head teacher* Relevant new policies or practices introduced *Focus group with peer supporters* Nature of support provided *Focus groups with peer supporters and MHFA trainees* Learning from the MHFA training Application of learning Whether and how learning shared with colleagues *All focus groups (intervention)* Any perceived changes in school ethos or activities *Interview with peer support service users* Whether the service helped and how
Reach	*Teacher questionnaires* Use of the peer support service MHFA training completed *Termly peer supporter meetings* Record of how much help provided *Focus group with peer supporters* How service has been promoted *Focus group with students (intervention)* Perceptions of support from teachers and any changes
Contamination	*Focus group with teachers (control)* Any mental health training Any perceived changes in school ethos or activities *Focus group with students (control)* Perceptions of support from teachers and any changes *Audit of school activities* Any new activities in control schools similar to intervention
Intervention fidelity	*Focus groups with peer supporters and MHFA trainees* How much ALGEE model is used when supporting others Other MHFA techniques used when supporting others *Training observations* How well trainer sticks to core MHFA components *Interviews with trainers* Perceptions of MHFA core elements
Unintended harm	*Termly peer supporter meetings* Record of any harm experienced/observed by peer supporters *Focus group with MHFA trainees* Any negative experiences of attending the training
Acceptability	*Interviews with head teachers (intervention)* Views on peer support service and MHFA training *Focus groups and interviews with non-peer supporter teachers (intervention)* Views/experiences of the peer support service *Training observations* Reactions of trainees to the training *Interview with trainers* What went well or not well in the training
Sustainability	*Interviews with head teachers (intervention)* What is needed to make the peer support service sustainable Ways to embed the MHFA learning *Focus groups with peer supporters* What is needed to make the peer support service sustainable *Audit of school activities (intervention)* Any wider changes e.g., policies, processes *Interviews with funders* How well the intervention fits with local priorities What is needed for the intervention to be rolled out

The teacher and student surveys will contain questions that relate to mechanisms of change and reach:

#### Mechanisms of change

Participants will be asked to rate stress and satisfaction at work, help provided and received at school, school’s attitude to staff and student wellbeing, and quality of relationships in school in the questionnaires, using Likert scales. Logistic regression models will be used to examine differences by study arm at follow up, adjusted for baseline scores, school-level FSM and geographical area. We will examine whether baseline measures of these variables moderate the effect of the intervention by including the appropriate terms in the analysis model – however this is purely an exploratory analysis as power will be low, and there is always the potential for unmeasured confounding of the association between moderator/mediator and outcome. We will also examine the extent to which the MHFA training appears to have impacted on mental health, wellbeing and helping behaviour. We will use a Complier Average Causal Effect (CACE) approach (using Instrumental Variable analysis or Principal Stratification) [48] to compare those who completed the training in the intervention schools with those in the control schools who would have completed the training, had they been offered it (based on matching their gender, role in the school and years of experience with those who were trained).

#### Reach

Follow up teacher surveys in the intervention schools will ask about use of the peer support service and participation in the MHFA training.

In addition, the following qualitative data will be collected:

- Interviews with the head teacher and an audit at each school at baseline and two year follow up to explore the school’s current activities in relation to mental health and wellbeing, any existing mental health improvement activities or plans and sustainability of the intervention.
- Termly meetings with the peer supporters to discuss service use and any harms.
- Observations of the training and interviews with the MHFA trainers to examine fidelity and acceptability.
- In four case study intervention schools the following will be held: focus group discussions with peer supporters, attendees of the MHFA for Schools and Colleges training, potential users of the peer support service, and year 9 students, and interviews with teachers who have used the peer support service. Topic guides will explore learning from the training and application of learning, acceptability and reach of the intervention, potential for harm, and potential for sustainability.
- In four case study control schools focus group discussions will be held with teachers and year 9 students to check for contamination, and examine usual practice.

One case study intervention school will be selected from each geographical area, ensuring contrasts between the four in terms of size and FSM eligibility. Case study control schools will be selected according to those that best match the case study intervention schools on these factors.

All qualitative data will be analysed thematically. Separate frameworks of codes for each ‘type’ of data will initially be constructed. As themes emerge these will be tested against new data and the frameworks modified until all data are accounted for. Once the frameworks are complete, common and contrasting themes across the different data ‘types’ will be examined. Transcripts of focus groups and interviews will be entered into the software package NVivo version 10, which will be used as a data management tool, permitting quick access to data that falls within each theme. The themes pertinent to the mechanisms of change; contextual influences; reach; contamination; intervention fidelity; unintended harms; acceptability; and sustainability will be integrated and used to construct interpretation of the study’s impact on the main outcomes. These findings may be used to inform post hoc secondary analysis of the outcomes, for example through creating thresholds for implementation, and examining whether high or low implementation affects the outcomes.

### Economic evaluation

We will prospectively record information on all MHFA training sessions (e.g., duration, trainers and trainees attending), including expenses claimed by trainers and incurred by schools (e.g., teaching cover, travel and venue hire). The costs of teacher time to receive MHFA training will be calculated based on national teacher pay scales. We will capture periodic ‘snapshots’ of peer support activity through termly feedback meetings, in which peer supporters will complete questionnaires regarding the previous 2 weeks’ support given.

We will examine whether these initial costs are offset by downstream savings (discounted at 3.5 % after 12 months) due to reduced absenteeism during the follow up period using human capital (i.e., salary) measures and alternative methods that take into account the broader impact on the school of teacher absences and or resignation/retirement [49]. The economic evaluation will be a cost consequence study from the public sector perspective estimating whether the incremental costs of the intervention are justified by improved teacher or student wellbeing, mental health, or performance. Because the potential benefits of the intervention are multifaceted and may affect others not directly observed in the study (e.g., students in other years), we believe a cost consequence framework is appropriate rather than a more reductive cost-effectiveness analysis which attempts to summarise efficiency in a single ratio [50].

We will estimate the incremental cost (and bootstrapped 95 % confidence interval) at the cluster-level, adjusting for school-level FSM and geographic area as stratifying variables. We will explore the economic consequences of the different models of MHFA training delivery used in England and Wales.

**Fig. 2:**
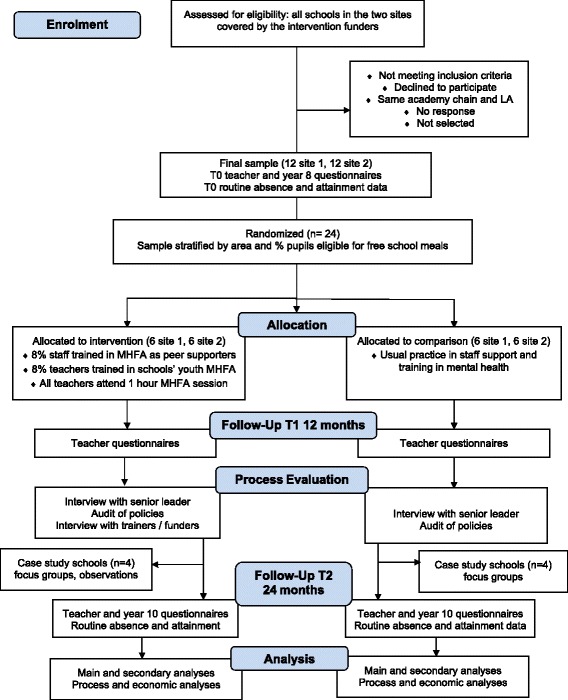
Schedule of data collection

The schedule of enrolment, allocation, data collection and analysis is shown in Fig. 2.

### Data monitoring

The study does not have a data monitoring committee as the risks of harm are small and effectiveness will not be able to be determined until the study end so no earlier stopping points are anticipated.

### Monitoring safety

School contacts and those delivering the intervention will be asked to contact the study team within two working days if any untoward incident or adverse event (AE) occurs to a member of staff or a student i) as a direct result of taking part in the WISE study or ii) because of changes that have occurred in the school environment due to participation in WISE. In these cases, study specific adverse event/incident forms will be completed, recording information on the event. Members of the study teams in Bristol and Cardiff will also be required to complete a form about any incidents or AEs that they encounter during data collections. All adverse event/incident report forms will be discussed with the Principal Investigator to assess seriousness and to explore causality. All AEs deemed to be ‘serious’ (SAE) – for example events which result in death or are life-threatening - will be reported to the Sponsor within 24 h. Where the SAE is suspected to be related to the intervention and unexpected, in other words a suspected unexpected serious adverse reaction (SUSAR), the Chair of the Trial Steering Committee (TSC) and the Ethics Committee will be notified in writing within 15 days of the study team receiving the initial report. An SAE which is not deemed to be related to the research will be reported to the TSC at the next scheduled meeting.

### Protocol changes

Any important changes to the protocol will be reported to the funders, the ISRCTN registry, the TSC and the ethics committee.

### Dissemination

A report will be prepared for each participating school containing the overall findings, and also each school’s anonymised school-level results from the teacher and student questionnaires. A full report of the study will be published in the NIHR Public Health Research journal and on the study website, and papers reporting on the main outcomes, as well as baseline, process evaluation and economic evaluation findings will be published in peer reviewed journals. If the findings show the intervention to be effective the intervention will be written up as a guidance document for schools, which will be disseminated via practitioner networks.

## Discussion

Despite evidence that teachers are at risk of poor mental health, and that teacher mental health can impact on student health and academic outcomes, no RCTs have focused on improving teacher wellbeing and mental health, alongside that of students. This full-scale cluster RCT tests the effectiveness of an intervention that has been shown to be feasible and acceptable in a pilot RCT. In addition, this trial will provide important information regarding the cost-effectiveness of the intervention, any implementation issues, and the extent to which it is likely to be sustainable.

## Additional files

Additional file 1: Participating schools research agreement. (DOC 217 kb)
Additional file 2: Parent survey information. (DOC 179 kb)
Additional file 3: Consent form for focus groups. (DOCX 56 kb)

## Abbreviations

AE: Adverse event
CACE: Complier average causal effect
FSM: Free school meals
MHFA: Mental health first aid
PHQ-8: Patient health questionnaire – 8 item
RCT: Randomised controlled trial
SAE: Serious adverse event
SDQ: Strengths and difficulties questionnaire
SUSAR: Suspected unexpected serious adverse reaction
TSC: Trial steering committee
WEMWBS: Warwick Edinburgh Mental Wellbeing Scale
WISE: Wellbeing in secondary education
