# Supplementary material for: Protocol for a cluster randomised controlled trial of an intervention to improve the mental health support and training available to secondary school teachers – the WISE (Wellbeing in Secondary Education) study
Source: BMC Public Health. 2016 Oct 18;16:1089. doi: 10.1186/s12889-016-3756-8 (PMC5070146; doi:10.1186/s12889-016-3756-8)
Supplement: Additional file 1: — Participating schools research agreement. (DOC 217 kb) [file 12889_2016_3756_MOESM1_ESM.doc]

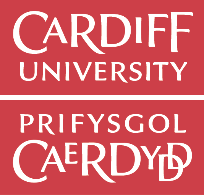

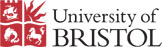
 **
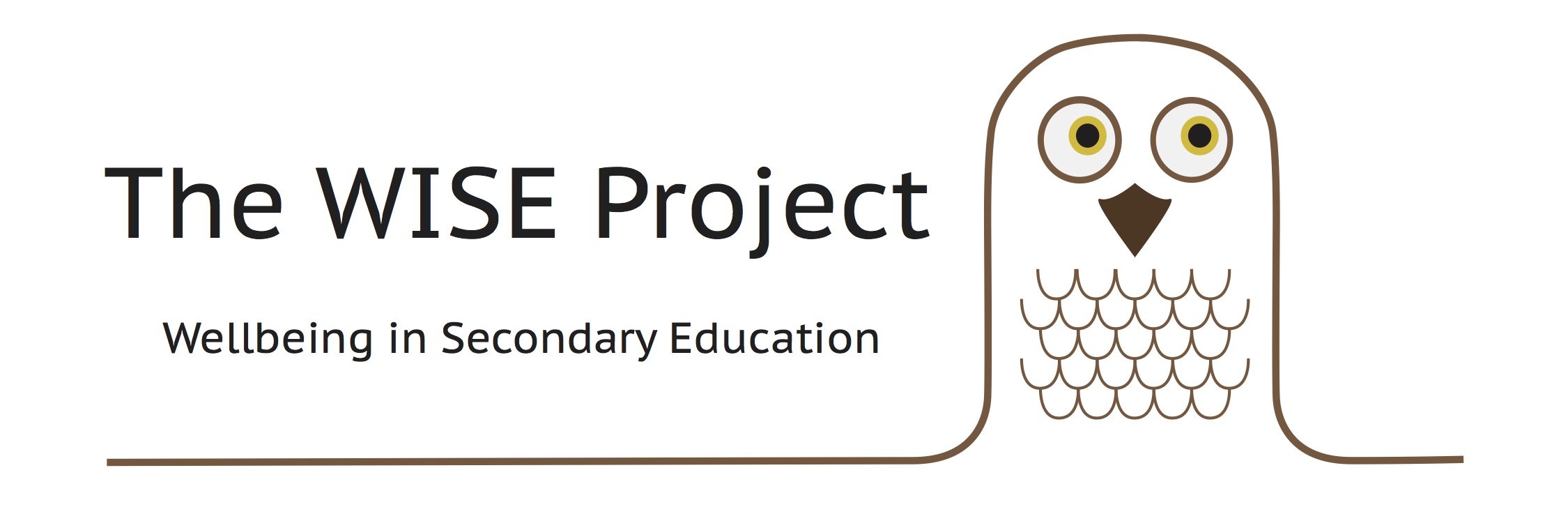
**

**RESEARCH AGREEMENT**

For the purposes of the study entitled **“A cluster randomised controlled trial of an intervention to improve the mental health support and training available to secondary school teachers – the WISE (Wellbeing in Secondary Education) project”** funded by the **National Institute of Health Research (England), Bristol City Council, Public Health England and Public Health Wales.**

This agreement dated ­­­________________ is made between:

The WISE study team, led by Dr Judi Kidger who is based at University of Bristol, School of Social and Community Medicine, Canynge Hall, 39, Whatley Road, Bristol, BS8 2PS.

AND

**[school name]** **[school address]**

**IT IS AGREED AS FOLLOWS,**

**1 Commitment from the study team**

The study team will

- Work with schools to identify the most convenient times to administer teacher and student surveys, and to run interviews and focus groups
- Cover postage costs for parent consent letters for the student surveys and focus groups
- Provide a study team member to administer each survey data collection session
- Provide sources of help sheets to everyone who completes a survey
- Pass information to schools in the event that a student writes something on a survey or says something during a focus group indicating they are at risk of harm
- In the intervention schools, liaise between schools and trainers to arrange the MHFA training, and cover the cost for the MHFA trainer. The study team will also reimburse schools for the cost of cover for teaching staff who attend the two day standard MHFA training on receipt of an invoice
- In the schools that do not receive the MHFA training (the comparison group) provide a thank you payment of £1000 (roughly the cost of a two day MHFA training course). Payment will be made once 80% of teacher surveys have been returned at the second follow up, on receipt of an invoice
- Report back anonymised school-specific data from the teacher and student surveys at the end of the final survey period
- Disseminate a final report about the study findings to all participating schools, and be available for oral feedback about the findings if schools request this (e.g. at governors’ or staff meetings)
- Anonymise all published data from the study, so no schools or individuals can be identified from any reports

**2 Commitment from the schools**

All participating schools will

- Provide a contact in the school to liaise with the research team and co-ordinate all research activities
- Identify approximately 30 minutes of meeting or training time during which the study team will collect survey data from all teachers in the summer terms of 2016, 2017 and 2018. It is vital that all teachers are available for this time – this can be done over more than one visit if necessary to accommodate part time workers
- Identify approximately 30 minutes of class or assembly time during which the study team will collect survey data from all year 8 students in the summer term of 2016, and from all year 10 students in the summer term of 2018. This can be done over several visits. A teacher must be present at all data collections to assist in keeping control and to comply with child protection requirements, but will not be asked to assist in the data collection
- Provide the research team with a list of staff and student names prior to each survey to allow the allocation of ID numbers
- Post or email consent letters home to parents of all students completing a questionnaire and inform the study team of any withdrawal of consent by parents (postage covered by the study team)
- Allow the research team to interview a member of senior management and – in a subsample of schools only – to conduct one focus group with a random selection of 6-8 teachers and one focus group with a random selection of 6-8 year 9 students about wellbeing in schools
- Allow the research team to gather information about the school’s policies and procedures relating to staff and student wellbeing at the beginning and end of the evaluation through examining documents and speaking to senior management as necessary
- Provide the research team with anonymised data regarding number of days of staff absence and pay grade of absent staff for the academic years 2015/2016 and 2017/2018
- Be randomly allocated to either receive the WISE intervention (intervention group) or to act as a comparison school (comparison group) for the evaluation of this work. This allocation will take place in the summer term of 2016, after the first surveys have been completed
- Accept the outcome of this allocation procedure. This means those schools allocated to the comparison group will not make their own arrangements to receive the intervention during the study period, and those allocated to the intervention group will commit to the extra tasks outlined below under “additional commitment from the intervention schools”

**3 Additional commitment from the intervention schools**

All schools allocated to the intervention group will

- Release a minimum of 8% of staff (half teaching, half non-teaching) to receive 2 days training in standard Mental Health First Aid (MHFA). Staff will be those who have received the most nominations from colleagues via the staff baseline survey, ensuring a range of gender, role and experience
- Allow staff trained in standard MHFA to deliver a confidential peer support service to colleagues, at least until the end of the academic year 2017/2018, and support any requirements for the smooth running of this service (e.g. allowance of time for planning meetings, opportunities to advertise the service during staff meetings and at the start of the academic year etc.)
- Commit a full INSET day between September to December 2016 for a minimum of 16 teachers to receive training in MHFA for Schools and Colleges
- Allow all teaching staff to receive a one hour introduction to mental health
- Allow the peer supporters an hour once a term to meet with the research team to discuss how the service is going
- In a subsample of schools only - allow the research team to hold 2 focus groups with the peer supporters, 2 focus groups with 6-8 of teachers who have received the youth MHFA training, 1 focus group with 6-8 teachers who have not had any training, 1 focus group with 6-8 year 9 students and interviews with approximately 5 staff who have used the peer support service between January 2017 and May 2018
- In a subsample of schools only - allow the research team to observe the MHFA training

AS AGREED BY:

**For and on behalf of:**

The WISE study team

Name:

Position:

Signature:

Date :

**For and on behalf of:**

**[school]**

Name:

Position:

Signature:

Date :
