# Supplementary material for: Protocol for a cluster randomised controlled trial of an intervention to improve the mental health support and training available to secondary school teachers – the WISE (Wellbeing in Secondary Education) study
Source: BMC Public Health. 2016 Oct 18;16:1089. doi: 10.1186/s12889-016-3756-8 (PMC5070146; doi:10.1186/s12889-016-3756-8)
Supplement: Additional file 2: — Parent survey information. (DOC 179 kb) [file 12889_2016_3756_MOESM2_ESM.doc]

| 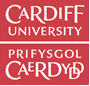 | 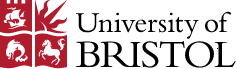 |
| --- | --- |
|  | |
|  | **School of Social and Community Medicine**  Canynge Hall, 39, Whatley Road, Bristol, BS8 2PS |
| **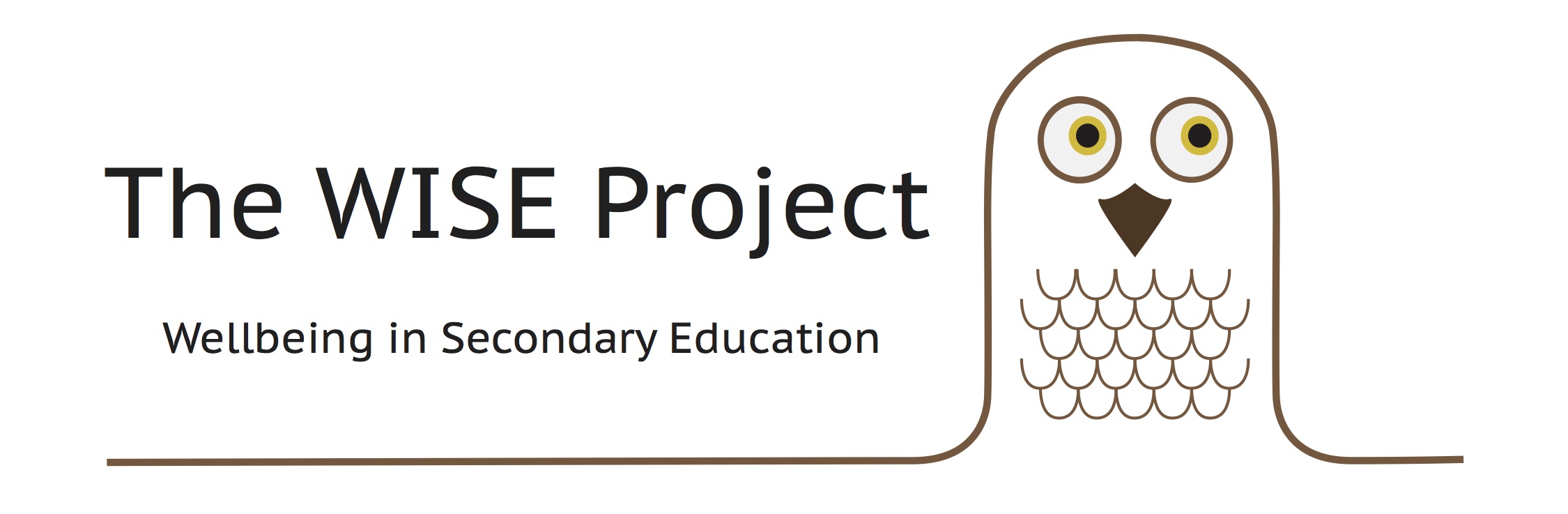** | **Dr Judi Kidger**  **Research Fellow**  T +44 (0)117 331 3910  F +44 (0)117 9287236  Judi.Kidger@bristol.ac.uk |
|  | |

Dear Parent/Guardian

Bristol and Cardiff Universities are running a research project looking at ways to improve the emotional health and wellbeing of teachers and students in secondary schools. We hope this will make schools better places for everybody. Your son or daughter’s school is one of 24 that are taking part in the study. In each participating school, students in year 8 will be invited to complete a short confidential survey, which includes questions about the support available to them at school, and some questions about their emotional health and wellbeing. The questionnaire will be repeated two years later. Your child can choose whether to complete the survey or not and is free to stop taking part at any time without giving a reason.

No one at the school will see what individual students have written: the research team will collect all the completed surveys, and will store them securely at the University. The questionnaires do not include names or other identifying features, just study ID numbers. The responses will be entered onto a database which is password protected, and only visible to the research team. We will share our findings with others who are interested, such as school communities, other academics, and policy makers, and we may publish them for example in academic journals. However, all the findings will be anonymised, so no schools or individuals will be able to be identified.

If you would like to know more about the project, or how we are going to use the information that we collect, please contact Sarah Bell [S.Bell@bristol.ac.uk](mailto:S.Bell@bristol.ac.uk). You may also use these contact details to find out the outcome of the study.

If you are happy for your son or daughter to complete the survey, then you do not need to do anything. If you **do not** want your son or daughter to take part in the survey, then please reply to this email address [odell.harriss@bristol.ac.uk](mailto:odell.harriss@bristol.ac.uk) stating your child’s name and school and stating “parental consent not given”, within one week of receiving this email.

Many thanks

Judi Kidger
