# Supplementary material for: Protocol for a cluster randomised controlled trial of an intervention to improve the mental health support and training available to secondary school teachers – the WISE (Wellbeing in Secondary Education) study
Source: BMC Public Health. 2016 Oct 18;16:1089. doi: 10.1186/s12889-016-3756-8 (PMC5070146; doi:10.1186/s12889-016-3756-8)
Supplement: Additional file 3: — Consent form for focus groups. (DOCX 56 kb) [file 12889_2016_3756_MOESM3_ESM.docx]

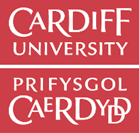

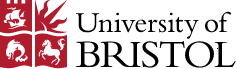


**The WISE project**

**
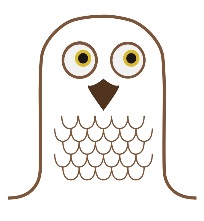
**

**PARTICIPANT CONSENT FORM FOR FOCUS GROUPS / INTERVIEWS**

|  |  | *Please initial* |
| --- | --- | --- |
| I have read and understood the information leaflet and have had the opportunity to ask questions |  | __________ |
| I understand that my participation is voluntary, and I am free to stop at any time without giving a reason |  | __________ |
| I consent to the focus group/interview being recorded |  | __________ |
| I understand the recording and transcript will be stored securely and used in the write up of the project |  | __________ |
| I agree to take part in the focus group / interview |  | __________ |

*Name* _____________________________________________

*Signature* _____________________________________________

*Date* _____________________________________________

*School* _____________________________________________________
